# Conventional, high frequency and differential targeted multiplexed spinal cord stimulation in experimental painful diabetic peripheral neuropathy: Pain behavior and role of the central inflammatory balance

**DOI:** 10.1177/17448069231193368

**Published:** 2023-09-15

**Authors:** Thomas J. de Geus, Glenn Franken, Elbert A Joosten

**Affiliations:** 1 168089 Department of Translational Neuroscience, School of Mental Health and Neuroscience, Maastricht University, Maastricht, Netherlands.; 2 199236 Department of Anesthesiology and Pain Medicine, Maastricht University Medical Centre, Maastricht, Netherlands.

**Keywords:** Spinal cord stimulation, painful diabetic peripheral neuropathy, inflammation, Glial cells, stimulation paradigms, differential target multiplexed

## Abstract

Spinal cord stimulation (SCS) is a last resort treatment for pain relief in painful diabetic peripheral neuropathy (PDPN) patients. However, the effectivity of SCS in PDPN is limited. New SCS paradigms such as high frequency (HF) and differential target multiplexed (DTM) might improve responder rates and efficacy of SCS-induced analgesia in PDPN patients, and are suggested to modulate the inflammatory balance and glial response in the spinal dorsal horn. The aim of this study was to research the effects of Con-, HF- and DTM-SCS on pain behavior and the spinal inflammatory balance in an animal model of PDPN. Streptozotocin-induced PDPN animals were stimulated for 48 hours with either Con-SCS (50Hz), HF-SCS (1200Hz) or DTM-SCS (combination of Con- and HF-SCS). Mechanical hypersensitivity was assessed using Von Frey (VF) test and the motivational aspects of pain were assessed using the mechanical conflict avoidance system (MCAS). The inflammatory balance and glial response were analyzed in the dorsal spinal cord based on RNA expression of pro- and anti-inflammatory cytokines (Tnf-α, Il-1ß, Il-4, Il-10), a microglia marker (Itgam), an astrocyte marker (Gfap), a T-cell marker (Cd3d), microglia proliferation markers (Irf8, Adgre1) and P2X4, p13-MAPK, BDNF signaling markers (P2x4, Mapk14, Bdnf). The results show that Con-, HF-, and DTM-SCS significantly decreased hypersensitivity after 48 hours of stimulation compared to Sham-SCS in PDPN animals, but at the same time did not affect escape latency in the MCAS. At the molecular level, Con-SCS resulted in a significant increase in spinal pro-inflammatory cytokine Tnf-α after 48 hours compared to DTM-SCS and Sham-SCS. In summary, Con-SCS showed a shift of the inflammatory balance towards a pro-inflammatory state whilst HF- and DTM-SCS shifted the balance towards an anti-inflammatory state. These findings suggest that the underlying mechanism of Con-SCS induced pain relief in PDPN differs from that induced by HF- and DTM-SCS.

## Introduction

Painful diabetic peripheral neuropathy (PDPN) is a common and chronic complication of Diabetes Mellitus (DM), affecting up to 25% of people with the disease.^1,2^ PDPN is characterized by neuropathic pain that starts as numbness in the feet and legs and significantly impacts a person's quality of life.^3^ Traditional pharmacological treatments for PDPN often have limited effectiveness, which urges the need and search for alternative therapies.^4^ Spinal cord stimulation (SCS) is such an alternative treatment option, and has been shown to be an effective therapy for PDPN patients.^5–10^ The use of SCS with conventional settings (conventional SCS [Con-SCS]) typically results in approximately 50% pain reduction in 50% to 70% of PDPN patients.^11^ Besides Con-SCS, new SCS paradigms like High-Frequency (HF) and Differential Target Multiplexed (DTM)-SCS have recently emereged.^12,13^ A clinical study has been performed using HF SCS in PDPN patients which showed promising result.^14^ DTM-SCS could potentially further improve pain reduction and responder rates.

The exact mechanism by which SCS provides pain relief in PDPN is not fully understood, but the activation of Aβ fibers in the dorsal columns with Con-SCS is known to exert both antidromic (mainly via local spinal γ‐aminobutyric acid (GABA)) and orthodromic-(mainly via descending serotonergic projections) effects.^11^ Furthermore, Con-SCS has been shown to modulate the activation of glial cells and expression of inflammatory molecules that might reduce pain.^13,15^ Animal studies using peripheral nerve injury (PNI) models to mimic chronic neuropathic pain have reported an activation of microglial and astroglial cells in the spinal dorsal horn upon injury.^16,17^ Activated microglia are known to release pro-inflammatory cytokines, which in a healthy state is typically followed by the release of anti-inflammatory cytokines to counteract the pro-inflammatory response and restore the central inflammatory balance.^18,19^ With chronic neuropathic pain the increase in pro- and at the same time decrease in anti-inflammatory molecules often lasts, creating a long-lasting inflammatory imbalance.^20–23^ SCS may reduce microglial and astrocyte activity and thereby restore the central inflammatory balance.^13,23,24^ In this regard, the recently developed DTM-SCS paradigm is suggested to specifically target the local inflammatory response in the spinal dorsal horn of chronic neuropathic PNI animals and restore the inflammatory imbalance.^21^ Besides DTM-SCS, the use of HF-SCS with a stimulation frequency of 1200Hz has also been shown to restore the inflammatory imbalance in the spinal cord of chronic neuropathic animals, albeit to a lesser extent.^13^ Described research was performed in PNI models. It is important to note that an increased pro-inflammatory state is also present in PDPN animal models.^25,26^ Therefore, HF- and especially DTM-SCS could potentially restore this balance and hereby further improve pain reduction in PDPN.

At a molecular level, it is known that various inflammatory molecules closely enhance the process of central sensitization and with that affect the sensitivity to painful stimuli.^18,27,28^ Moreover, it has been shown that in an animal model of chronic neuropathic pain after PNI the expression of pro-inflammatory cytokines such as TNF-α and IL-1β is increased in the spinal dorsal horn, while the expression of anti-inflammatory cytokines such as Il-4 and Il-10 is decreased.^28,29^ In a similar PNI model, activated microglia cells are also known to release various neurotrophins including Brain Derived Neurotrophic Factor (BDNF).^30,31^ In more detail, activation of purinergic P2X4R receptors, which are abundantly present in microglial cells in the spinal dorsal horn, results in increased secretion of BDNF via activation of p38-MAPK. BDNF has been linked to central sensitization and increased pain behavior in chronic neuropathic animals, specifically in males.^32,33^ Furthermore, in the spinal cord, alterations in gene levels of markers for microglial proliferation (*Erm1)* and transformation towards a reactive phenotype (*Irm8)* in a PNI chronic neuropathic pain model have been reported.^34,35^ In contrast, development of chronic neuropathic pain in females has been mainly linked to activation of T cells in the spinal dorsal horn.^33^

To date, the effect of different SCS paradigms on the central spinal inflammatory balance and its corresponding inflammatory molecules in PDPN animals has not been investigated. Therefore, the present study aimed to investigate and compare the effect and role of various SCS paradigms (Con-, HF- and DTM-SCS) on the spinal inflammatory response and pain relief in an animal model of PDPN. Pain behavior was assessed with use of the Von Frey (VF) paw withdrawal reflex test to asses mechanical hypersensitivity as well the operant Mechanical Conflict Avoidance System (MCAS) to assess the motivational aspect of pain.^36^ The effect of the various SCS paradigms on the spinal inflammatory balance was analyzed based on RNA expression of pro inflammatory cytokines (*Tnf-α*, *Il-1ß*), anti-inflammatory cytokines (*Il-4, Il-10*), a microglia marker (*Itgam*), an astrocyte marker (*Gfap*), a T-cell marker (*Cd3d*), microglia proliferation markers (*Irf8*, *Adgre1*) and P2X4, p13-MAPK, BDNF signaling markers (*P2x4, Mapk14, Bdnf*).

We hypothesized that Con, HF and to an ever higher extend DTM-SCS result in pain relief, reflected in a decrease in sensitivity (VF) and in a lower exit time on the MCAS. Furthermore, we expect HF and DTM results in a restoration of the spinal inflammatory balance, whilst Con SCS might not or further disrupt this balance.

## Methods

### Ethics statement

The Animal Research Committee of the Maastricht University Medical Centre (under project license 2017–022) approved the experiments as described in this study. All experiments were performed in accordance with the guidelines of the European Directive for the Protection of Vertebrate Animals Used for Experimental and Other Scientific Purposes (86/609/EU).

### Animals

The experiments were performed using young adult female Sprague Dawley rats (n=64), aged 5 weeks at the start of the experiment. Animals were randomly housed in groups of two in polycarbonate cages in a climate-controlled vivarium (temperature 21 ± 1°C, humidity 55 ± 15%). Animals were housed at a 12/12 reversed day-night cycle with ad libitum access to food and drinking water. Experiments were performed between 8:00h and 18:00h, which is during the night cycle when rodents are more active than during day light.

### Experimental design

**Figure 1.**
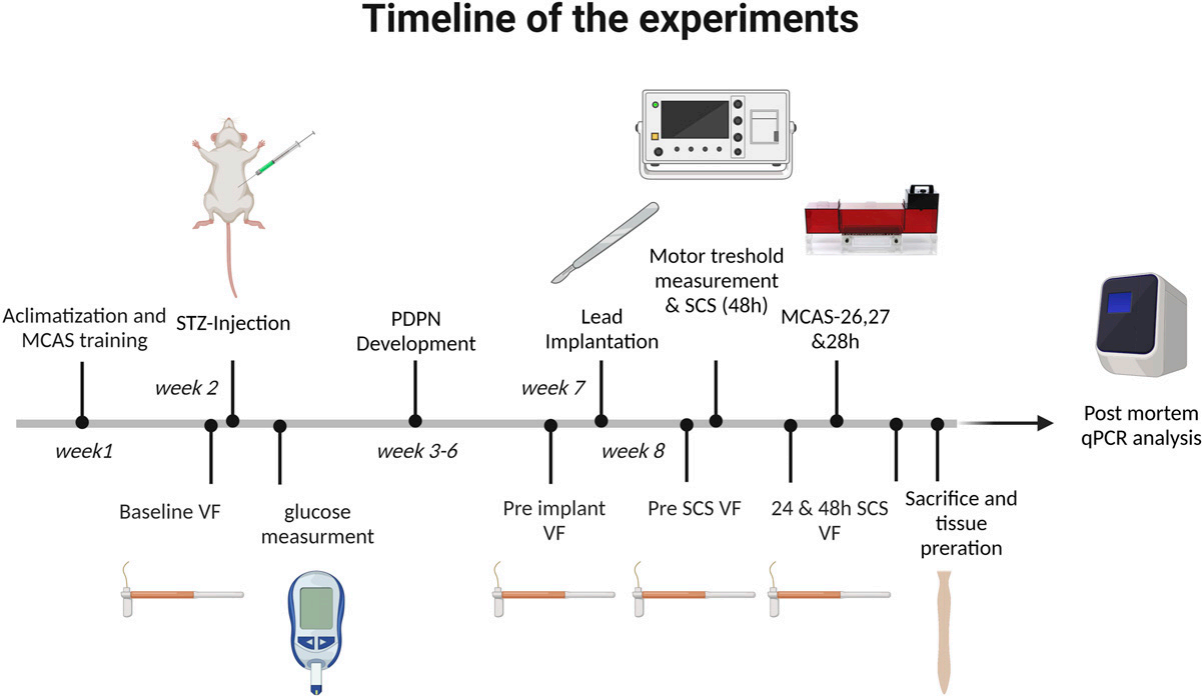
Timeline of the experiments.

After acclimatization to the housing facility, animals were familiarized with the VF setup before VF-measurement and/or trained for the MCAS.^36,37^ Before STZ injection, baseline PWT was measured using VF. Five days after STZ injection, blood glucose levels were measured and only diabetic animals (blood glucose >15 mmol/L) were kept in the experiment. Four weeks after STZ injection, mechanical hypersensitivity (VF) was measured in diabetic animals. Animals with a drop of ≥0.2 in log_10_ 10000x 50% PWT were implanted with a 4-contact SCS lead. After 3 days recovery, the pre-SCS PWT was measured. Next, the MT was determined and the animals were randomized (using randomize.org), to either Sham-, Con-, HF- or DTM-SCS. The researcher was blinded when assessing PWT or MCAS which was concealed from the assessing researcher throughout the experiment. Thereafter, the stimulation was turned on. After 24 and 48 hours of continuous SCS, PWT was measured using VF. After the 24 hour VF-measurement the MT was again determined and amplitude was adjusted accordingly to achieve stimulation at 50%MT. Operant pain testing with use of MCAS was performed after 26,27 and 28 hours of SCS, during which the animals needed to be decoupled from the stimulation setup. After the last VF measurement, animals were sacrificed using an overdose pentobarbital followed by transcardial perfusion with Tyrode-buffer. Lastly, tissue was extracted and prepared for qPCR analysis as described. A time line of the experiments is provided in Figure 1.

### Induction of diabetes mellitus

Induction of DM in Sprague-Dawley rats was performed as described before.^38,39^ Briefly: animals were weighed and fasted overnight before they received a single intraperitoneal injection with 65 mg/kg streptozotocin (STZ) (Merck, Darmstadt. Germany) freshly dissolved in 0,9% NaCl in order to induce DM. Five days after STZ injection the blood glucose levels of the animals were measured using an Accu-Chek Aviva® glucometer (Roche Diagnostics GmbH, Mannheim, Germany). Animals with a blood glucose level of ≥ 15 mmol/L were considered diabetic and were included in the study.^40^ If glucose levels exceeded 31.4 mmol/L, one third of a slow releasing insulin pallet (LinShin Inc. Canada) was placed subcutaneously in the neck of the animal.

### Reflex mediated response and pain: assessment of mechanical hypersensitivity

Mechanical hypersensitivity was assessed by applying VF monofilaments (bending forces 0.4, 0.6, 1.2, 2.0, 3.6, 5.5, 8.5, 15.1, and 28.84 g) (Stoelting, Illinois. USA) to the plantar surface of the hind paws of the animals. Rats were individually placed in plastic cages with a mesh floor and allowed to acclimatize to the environment for 15 minutes prior to testing. The 50% paw withdrawal threshold (PWT) was calculated based on the up and down method.^41^ The calculated 50% PWT values were then multiplied by 10,000 and logarithmically transformed to obtain a linear scale and account for Weber’s law.^38,39,42,43^ Animals with a decrease of ≥0.2 in the log_10_ 10000X 50% PWT in any of the two hind paws were considered to have mechanical hypersensitivity and were implanted with an SCS electrode.

#### Operant response and pain: Mechanical Conflict Avoidance System

Rats underwent habituation and training to the MCAS as described in detail by Harte et al. 2016.^37^ All behavioral testing was performed in a quiet room where the animals also received SCS. The MCAS consists of a light compartment a probe compartment with nociceptive probes, and a dark compartment. Rats were individually placed into the light compartment with the light turned off. After 15 seconds of acclimatization, the light was turned on. Rats are photophobic and naturally want to escape from the light. Twenty seconds after the light was turned on, the door of the light compartment was opened. The exit latency is defined as the time between opening of the door of the light compartment and the rat placing all four paws on the probe compartment. When the animals crossed the nociceptive probes and reached the dark compartment with all four paws, the door of this compartment was closed and animals were returned to their cage after 20 seconds in the dark compartment. The time between the exit (or escape) from the light compartment and entering of the dark compartment was defined as the crossing duration. Rats that successfully escaped the light compartment but did not enter the dark compartment within 30 seconds hereafter were marked as “failed to cross” and returned to their cage until the next trail. Animals that did not leave the light compartment within 30 seconds after the light was turned on were marked as “failed to exit” and returned to their cage until the next trial. The test procedure was repeated three times after 26, 27 and 28 hours of SCS at a probe height of 4mm, as prior research showed significant differences in exit latency at this probe height during SCS in a neuropathic pain model.^36^ One hour prior to each first trial, rats underwent a trial without nociceptive probes in order to re-familiarize with the system.

### Implantation of spinal cord stimulation lead

A 4-contact custom-made cylindrical SCS lead (Oscor Inc. Palm Harbor. USA) was implanted epidurally according to standard protocol and connected to a connector block in the neck as performed by Vallejo and colleagues.^13,15^ In short, the spinal cord was exposed by lumbar incision and laminectomy of the L2 vertebrae. The dura was kept intact during the procedure. Next, the lead was rostrally introduced in the epidural space towards the L1-T13 vertebrae to cover L4-L5 spinal cord segments. The lead was anchored using Histoacryl (B. Braun, Rubi, Spain) and the lead was tunneled under the skin to the neck of the animal. The lumbar incision was closed in layers and the electrode was externally connected to a custom-made connector block which was attached to a rat jacket (Lomir Biomedical Inc, Quebec, Canada). Electrode configuration was set at alternating cathode and anode settings (rostral to caudal: + − + −).

### Spinal cord stimulation

For spinal cord stimulation (SCS) of the dorsal columns, a stimulator (DS8000-channel digital stimulation, World Precision Instruments, Sarasota, USA) connected to an isolator (DLS100) was used. The stimulation was set to deliver constant-current biphasic stimulation for all stimulation paradigms used in the study. The motor threshold (MT) for all paradigms was determined using a pulse-width of 150 µs administered at a frequency of 2Hz. The amplitude was gradually increased until contractions/twitches of the muscles in the hind limbs and/or lower trunk were perceived (either by feeling or observing) as shown in previous research.^38,39,43^ Animals received stimulation of the dorsal columns either via Con- SCS (frequency 50 Hz, 150 µs pulse with, 50%MT, on both electrode pairs), HF-SCS (frequency 1200 Hz, 50 µs pulse at 50%MT, on both electrode pairs), DTM-SCS (frequency 50Hz, 150 µs pulse at 50%MT, on the rostral electrode pair and frequency 1200 Hz, 50µs pulse at 50%MT, on the caudal electrode pairs), or Sham-SCS during which the amplitude was set to 0. All stimulation forms were applied using biphasic pulses with active recharge balancing. The animals were stimulated continuously for 48 hours. The MT of each individual animal was determined before stimulation and again checked for after 24h of stimulation.

### Tissue preparation

Following transcardial perfusion with Tyrode buffer (pH 7.4), the spinal cord was extracted via spinal flush as previously described by Richner et al.^44^ In short, spinal flush was performed by isolating the spinal column and flushing Tyrode trough the spinal canal from caudal towards the cranial direction using a 10mL syringe and 16 gauge needle. The spinal cord was then immediately cut and spinal levels L1-L6 were selected and the tissue was further sub-sectioned into 4 parts: left ventral, right ventral, left dorsal and right dorsal. Tissue was then stored until further use in RNA-later (Thermo-Fisher Scientific, Waltham (CA), USA) at -80^o^C.

### Real-time polymerase chain reaction

Spinal cord tissue was defrosted and homogenized using a T 10 basic ULTRA-TURRAX® (IKA, Germany). RNA was isolated using Trizol (Invitrogen, Waltham (CA), USA) and cDNA was synthesized using the MAXIMA H Minus cDNA Synthesis Master Mix (Thermo Fisher Scientific, Waltham (CA), USA), according to manufacturer’s instructions. Quantitative PCR (qPCR) was conducted on a CFX348 Thermal Cycler (Bio-Rad Laboratories, Hercules (CA), USA) using PowerTrack™ SYBR Green Master Mix (Applied Biosystems, Warrington, UK). Relative quantification of gene expression was accomplished by using the comparative 2^−ΔΔCT^ method using *Gapdh* as gene for normalization.^45^ The forward and reverse primers of the housekeeping and genes of interest are depicted in table 1.

**Table 1.** List of Used Primers and Their Corresponding Forward (F) and Reverse (R) Nucleotide Sequences.

Gene symbol	Gene Name	Forward and reverse primer sequence
*Adgre-1*	Adhesion G protein-coupled receptor E1	F: TTTGTGAATGCCACCTTTACC R: TGTAGCTGCACTCTGTAAGG
*Cd3d*	CD3 delta subunit of T-cell receptor complex	F: GTATATGTGTAATGGGACAGAGG R: CACAGTTCTGGCACATTCG
*Eef1a1*	Eukaryotic Translation Elongation Factor 1 Alpha 1 (housekeeping)	F: TGCTGGAGCCAAGTGCTAAT R: GTGCCAATGCCGCCAATTTT
*Gapdh*	Glyceraldehyde-3-phosphate dehydrogenase (Housekeeping)	F: AGACAGCCGCATCTTCTTGT R: TGATGGCAACAATGTCCACT
*Gfap*	Glial fibrillary acidic protein	F: AAATCTGTGTCAGAAGGCCA R: CTCCTTAATGACCTCGCCA
*Il-1ß*	interleukin 1 beta	F: CAGGAAGGCAGTGTCACTCA R: AAAGAAGGTGCTTGGGTCCT
*Il-10*	Interleukin 10	F: TGGCCCAGAAATCAAGGAG R: GAAATCGATGACAGCGTCG
*Il-4*	Interleukin 4	F: CAGGGTGCTTCGCAAATTTTAC R: CACCGAGAACCCCAGACTTG
*Irf-8*	Interferon regulatory factor 8	F: TAGACCCAACAAGCTGGAG R: AGAACTGCTGCAGGTCTC
*Itgam*	Integrin subunit alpha M	PrimePCR SYBR Green Assay: ITGAM (Bio-rad laboratories, Hercules (CA), USA)
*Mapk14*	Mitogen-activated protein kinase 14	F: AGATAATGCGTCTGACGGG R: ACTGAATGTAGTTTCTTGCCTC
*P2rx4*	Purinergic receptor P2X 4	F: CGTGGCGGACTATGTGATT R: GTGATGTTGGGGAGGATGTTC
*Tnf-α*	Tumor necrosis factor α	F: CTTCTCATTCCTGCTCGTG R: TTTGGGAACTTCTCCTCCT

## Statistical analysis

All data is presented as mean ± standard error of the mean (SEM). For statistical analysis of the VF-data overtime within group and per time point between groups a two-way analysis of variance (ANOVA) was performed, followed by Tukey’s multiple comparison test. For the comparison of the exit latency and crossing duration of the MCAS between groups a one-way ANOVA was used. Furthermore, for analysis of the RNA expression, the 2^−ΔΔCT^ values were log transformed. Next, the log2^−ΔΔCT^ of the groups were compared using a one-way ANOVA followed by Tukey’s multiple comparison if applicable. A *p* value of ≤0.05 was considered statistically significant. All statistical analysis were performed using GraphPad Prism software version 6.01 (GraphPad Software, Inc., San Diego, CA, USA).

## Results

### SCS-paradigms and pain behavior

#### Reflex mediated paw withdrawal response:

**Figure 2.**
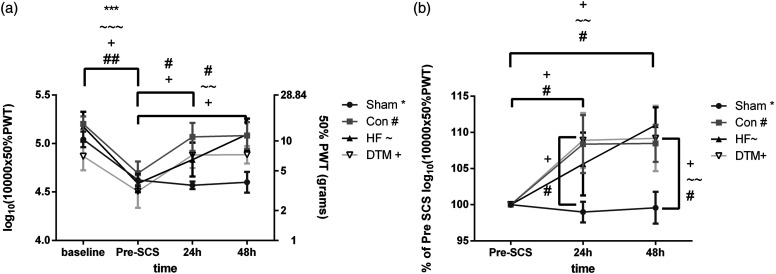
(a). Log10 10000X 50% PWT over time. Animals developed significant STZ-induced mechanical hypersensitivity from baseline to pre-SCS. Con-, HF- and DTM-SCS but not Sham-SCS significantly reduced hypersensitivity after 48 hours of continuous stimulation. (b). Log10 10000X 50% PWT as percentage of pre-SCS. Con-, HF- and DTM-SCS significantly increased Log10 10000X 50% PWT values as compared to sham after 48 hours of stimulation. Con, HF and DTM SCS do not significantly differ from each other. *, ∼,+,#*p* < 0,05; **, ∼∼,++,##*p* < 0,01; ***,∼∼∼,+++,###*p* < 0,001.

The mean log_10_ (10,000x 50% PWT) of the implanted animals did not differ over the groups and was: 5.0 ± 0.3 in animals assigned to sham (n = 10), 5.2 ± 0.2 in animals assigned to Con (n = 6), 5.2 ± 0.4 in animals assigned to HF (n = 6) and 4.9 ± 0.4 in animals assigned to DTM (n = 6) at pre-STZ baseline. The log_10_ 10000X 50% PWT dropped to 4.6 ± 0.2 (*p* = .0006*)*, 4.7 ± 0.3 (*p* = .0015), 4.6 ± 0.2 (*p* = .0002) and 4.5 ± 0.4 (*p* = .039), due to STZ induced DM, after implantation of the electrodes and before SCS in the Sham, Con, HF, and DTM group, respectively. After 24 hours of SCS, the log_10_ 10000X 50% PWT significantly increased in the Con and DTM group as compared to pre-SCS (*p* = .03 and *p* = .03, respectively), but not in the sham and HF group (*p* = .95 and *p* = .26, respectively). After 48 hours of continuous stimulation, the log_10_ 10000X 50% PWT in the Con-, HF- and DTM-SCS groups was significantly increased as compared to pre-SCS; *p* = .022, *p* = .0015 and *p* = .028 respectively (Figure 2(a)). Sham SCS did not result in an increase of the log_10_ 10000X 50% PWT after 48 hours (*p* = .99) (Figure 2(a)). In order to compare the effects of different SCS paradigms, Figure 2(b) shows the log_10_ 10000X 50% PWT as percentage of pre-SCS. The log_10_ 10000X 50% PWT is significantly increased as compared to Sham SCS both for Con-SCS (after 24 and 48 hours of stimulation (*p* = .034 (24h) and *p* = .023 (48H); and for DTM-SCS (after 24h *p* = .049 and 48 h. *p* = .02948h). Furthermore, the log_10_ 10000X 50% PWT is significantly increased in the HF group as compared to sham after 48hours of stimulation (*p* = .006).

#### Operant pain testing using MCAS

**Figure 3.**
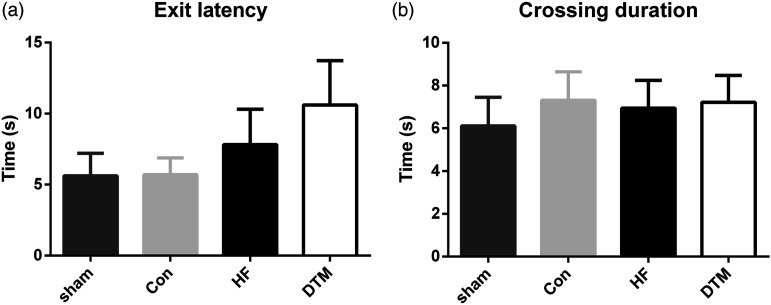
Average exit latency (a) and crossing duration (b) after 26, 27 and 28 hours of SCS on 4 mm probe height in the Mechanical Conflict Avoidance System for PDPN animals treated with Sham-, Con-, HFand DTM-SCS. There are no significant differences between the groups.

Behavioral analysis of the motivational aspects of pain using the MCAS showed no difference in exit latency nor crossing duration between any of the groups (see Figure 3).

### SCS-paradigms and RNA expression of pro- and anti-inflammatory cytokines in spinal dorsal horn

**Figure 4.**
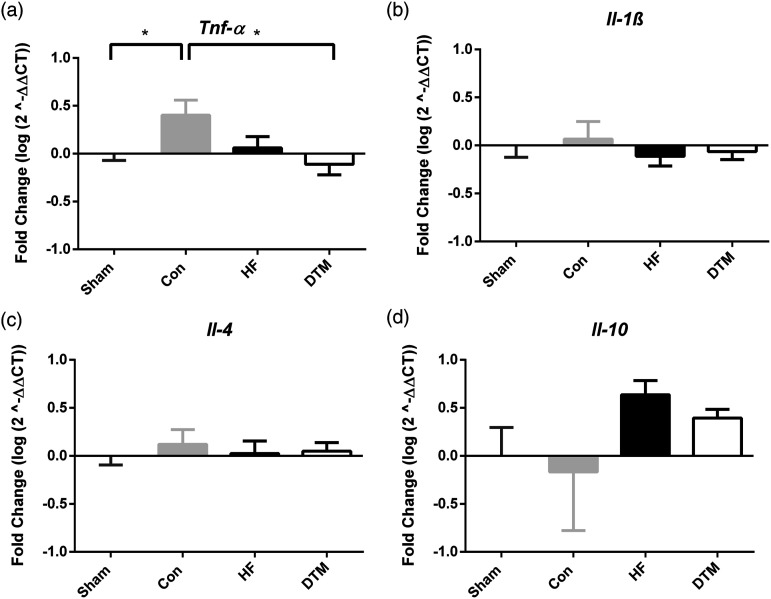
Effect of Sham-, Con-, HF- and DTM-SCS on relative RNA levels of Pro-inflammatory Tnf-α (a) and Il-1ß (b), and anti-inflammatory Il-4 (c) and Il-10 (d) in the spinal dorsal horn of PDPN animals. *p < 0.05.

**Figure 5.**
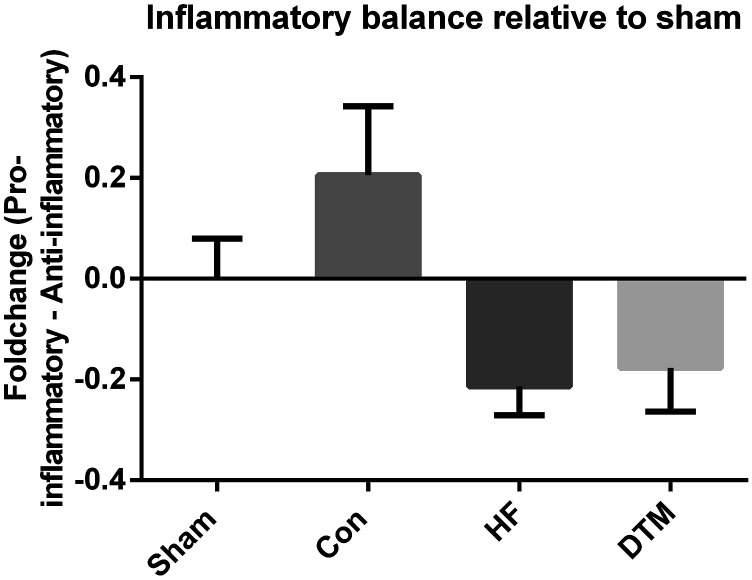
Impression of the effect on the balance between pro- and anti- inflammatory cytokines by Con-, HF and DTM- SCS, relative to Sham-SCS. Impression of the effect of Con-, HF- and DTM-SCS on relative RNA levels on balance of pro- and anti-inflammatory cytokines relative to Sham-SCS . The average of the log2-ΔΔCT of the anti-inflammatory cytokines was subtracted from the log2-ΔΔCT of the pro-inflammatory cytokines. A positive value indicates a shift towards more pro- and less anti-inflammatory cytokines and a negative value indicates a shift towards more anti- and less pro-inflammatory cytokines. As these experiments are limited to two pro- and two-anti-inflammatory cytokine markers, we did not perform any statistics on this analysis and is solely indicative. Con-SCS results in a balance towards more pro- and less anti-inflammatory cytokines, whilst HF- and DTM-SCS results in a balance towards less pro and more antiinflammatory cytokines.

Con-SCS did result in a significant increase in pro-inflammatory cytokine *Tnf-α* RNA expression after 48 hours as compared to Sham-SCS (*p* = .039) and DTM-SCS (*p* = .022) (Figure 4). RNA-expression of cytokines *Il-1ß*. *Il-4* and *Il-10* was not affected after stimulation with either of the SCS-paradigms (Con-SCS, HF-SCS or DTM-SCS). Visualization of the effects of Con-, HF- and DTM-SCS on the inflammatory balance compared to Sham-SCS suggests that Con-SCS results in an increase of pro- and decrease of anti-inflammatory markers, whilst HF- and DTM-SCS result in an opposite effect (Figure 5).

### SCS-paradigms and RNA expression of cell type and microglial proliferation markers in spinal dorsal horn

**Figure 6.**
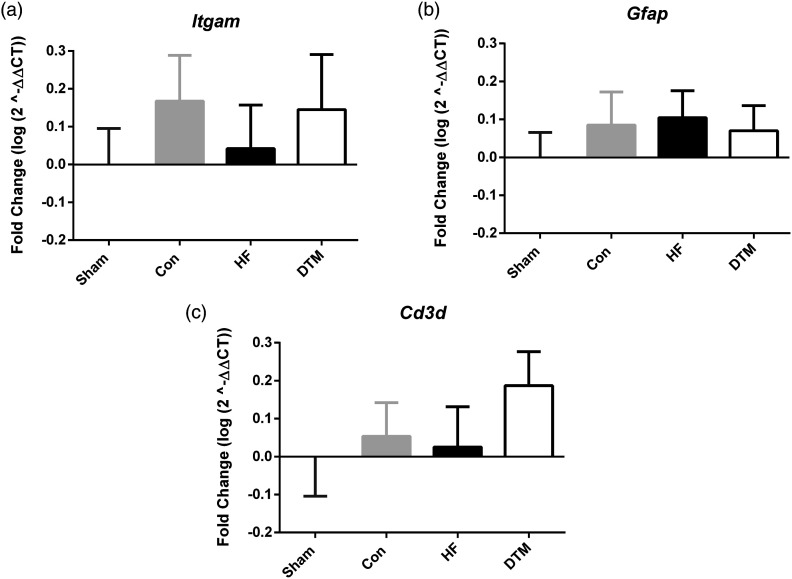
Effect of Sham, Con, HF- or DTM-SCS on relative RNA levels of microglial cell marker Itgam (a), Astrocyte marker Gfap (b), and T-cell marker Cd3d (c) in spinal dorsal horn of PDPN animals.

**Figure 7.**
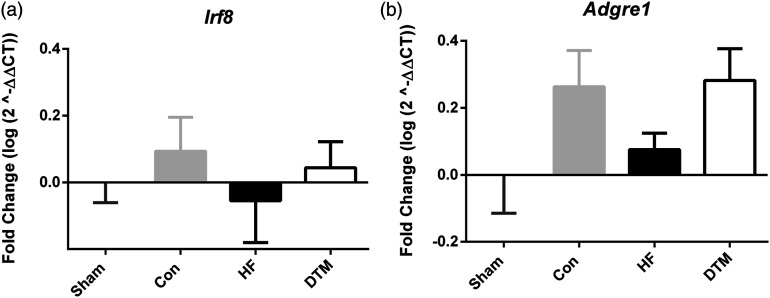
Effect of Sham, Con-, HF- and DTM-SCS on relative RNA levels of microglial proliferation markers Irf-8 (a) and Adgre-1 (b) in spinal dorsal horn of PDPN animals.

There are no significant differences in the RNA-expression of cell type markers for microglia (*Itgam*), astrocytes (*Gfap*) and T-cells (*Cd3d*) after 48 hours of Sham-, Con-, HF- and DTM-SCS (Figure 6). Furthermore, there is no significant difference noted with respect to the RNA expression of microglial proliferation markers *Irf-8* and *Adgre1* (Figure 7).

### SCS-paradigms and RNA expression of P2X4-MAPK pathway and neurotrophic factor BDNF markers in spinal dorsal horn

**Figure 8.**
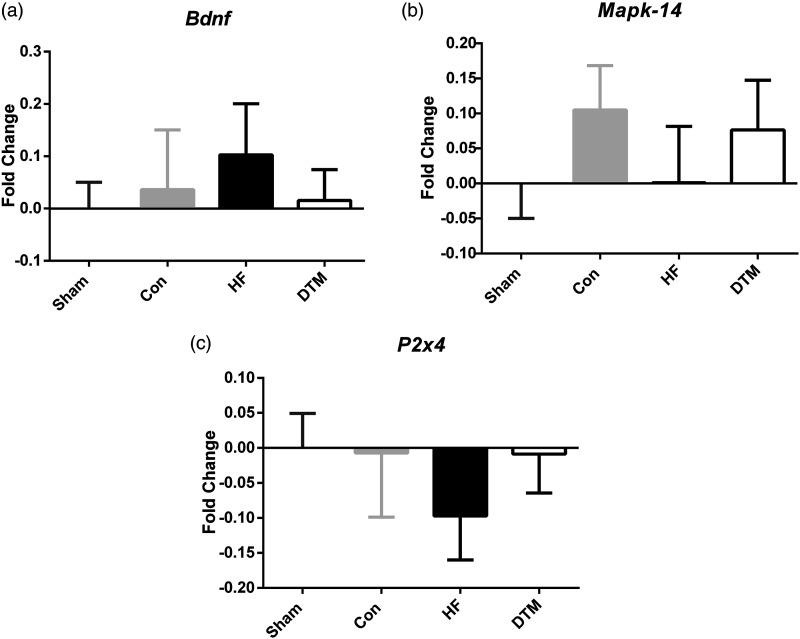
Effect of Sham, Con-, HF- and DTM- SCS on relative RNA levels of Bdnf (a), Mapk-14 (b), and P2x4 (c) in spinal dorsal horn PDPN animals.

No significant differences in RNA expression levels of *P2x4* and *Mapk14* between any of the SCS-paradigms and Sham-SCS is noted*.* Furthermore, also RNA expression of neurotropic factor *Bdnf* did not significantly differ between the SCS paradigms and sham-SCS (Figure 8).

## Discussion

From our experiments we conclude that: 1.Con-, HF-, and DTM-SCS are equally effective at reducing mechanical hypersensitivity in STZ-induced PDPN animals after 48h of continuous stimulation. 2. Motivational aspects of pain after 26 to 28 hours of stimulation were not affected by either Con-, HF- or DTM-SCS. 3. Con-SCS significant increases the pro-inflammatory cytokine TNF-alpha after 48 hours of stimulation as compared to Sham- and DTM-SCS. 4. Con-SCS seems to shift the inflammatory balance towards a pro-inflammatory state whilst HF and DTM shift this balance towards an anti-inflammatory state. 5. None of the SCS-paradigms showed an effect on the selected cell type markers for microglia, astroglia or T-lymfocytes nor markers for microglial proliferation, or the P2X4-BDNF pathway.

Behavioral pain research on the effects of DTM-SCS in neuropathic pain models showed DTM-SCS to be significantly better at reducing mechanical hypersensitivity as compared to Con- or HF-SCS.^13^ This research was performed in an PNI animal model of chronic neuropathic pain.^13^ The present study is based on a chronic neuropathic model due to induction of DM. The differences in pathophysiology between the models might underlie the observed difference, although it should be taken into account that both in PNI as well as in PDPN damage to peripheral nerves are a prominent feature involved in the development of chronic neuropathic pain. An alternative explanation for the differences between the findings in literature based on use of a PNI model of chronic neuropathic pain and our findings in a PDPN neuropathic pain model might be related to the sex used. In our study, female rats were used whereas in the PNI model studies male rats were used.^13^ A distinct mediation of pain hypersensitivity in rodents has previously been reported. Whilst in males microglia have a dominant role, in females t-cells possible mediate pain hypersensitivity.^33^ To our knowledge, it is not known if SCS might have differential effects or mechanisms in males versus females. Nevertheless, our results and effect size as noted with Con-SCS on mechanical hypersensitivity in PDPN animals is reproducible and identical as previously reported.^39,43^ Furthermore, previous research in PDPN animals showed no major difference in effect on mechanical hypersensitivity between Con (50Hz) and HF- (500Hz) SCS, which is in line with our findings.^43^ Furthermore, the chronification of pain might also be a factor. In most studies using PNI models, only 5 days after induction of the nerve injury and development of pain SCS was applied, whilst in this study 4 weeks after induction of DM SCS was applied.^13^ This might lead to a more chronic state of the pain in the PDPN animals and this is clinically more relevant. One could hypothesize that HF and DTM SCS interfere with the onset or development of the neuropathic pain. For this study all SCS waveforms were applied at 50% of the motor threshold to be able to compare the results with previous studies on DTM.^13^ As the main variable between the SCS-paradigms used is the frequency, other parameters (including intensity) were kept constant.

To our knowledge this is the first paper studying the effects of SCS-induced analgesia and operant testing (in the MCAS) in an animal model for PDPN. Meuwissen *et al.* investigated the effects of 30 minutes of Con-SCS or Burst-SCS in an animal model of PNI-induced chronic neuropathic pain on the escape latency and crossing duration in the MCAS.^36^ Their findings showed a decreased exit latency with use of both Con- (50Hz, 66%MT) and Burst-SCS(40Hz interburst, 449Hz intraburst, 50%MT) as compared to Sham-SCS. Furthermore, the study of Meuwissen and colleagues noted a significant difference in MCAS escape latency between Con- and Burst-SCS, suggesting that Burst SCS, more than Con-SCS affects the motivational aspect of pain. Based on these results in the PNI-model it was hypothesized that with use of Con-, as well as with HF- and DTM-SCS a reduction in exit latency would also occur in the PDPN model. As none of the SCS paradigms showed an effect on escape latency it is reasonable to suggest that this is related to the animal model used. PDPN is a systemic model and DM and its effect is not limited to the hind paws (in contrast to the PNI model), and thus the forepaws might be affected as well. This may have impact on the pain relief as the leads only cover spinal cord levels related to hind limb innervation. Furthermore, in our study animals were not decoupled from the stimulator for the time of MCAS testing as they were in the Meuwissen study.^36^ It therefore might be possible that the effects of SCS on the motivational aspects of pain do not have a wash out time, which would explain why we don’t see an effect with any of the paradigms tested.

Nevertheless, neither Con- nor HF- or DTM-SCS did affect MCAS escape latency in PDPN animals. This implies that the SCS-paradigms and pain relief in PDPN animals does not include motivational aspects of pain. Using both the reflex mediated von Frey as well as the operant test MCAS no differences were noted on “pain inhibition” between any of the paradigms. Nevertheless, this does not exclude the possibility of differential effects of the various SCS paradigms on other aspects of pain as for instance spontaneous pain or thermal hyperalgesia.

A recent review highlighted distinct effects of various SCS paradigms on the inflammatory balance in animals for neuropathic pain.^20^ In general, Con-SCS results in an increased inflammatory imbalance whilst HF and especially DTM-SCS tend to restore this imbalance. Our results indicate that Con-SCS leads to a significant increase of *Tnf-α* as compared to Sham- and DTM-SCS. This effect is in line with the results previously described.^28,46^ TNF*-α* is reported to increase synaptic strength, by among others phosphorylation of NMDA receptors.^46^ The latter induces central sensitization, known to be a pivotal process in development of neuropathic pain.

On the other hand pro-inflammatory marker *IL-1b* and anti-inflammatory marker *Il-4* and *Il-10* did not significantly change after any of the SCS paradigms tested. Nevertheless, a pattern is observed as visualized in Figure 5 on relative RNA levels in the balance of pro- and anti-inflammatory cytokines. Con-SCS tends to shifts the inflammatory balance towards a pro-inflammatory state whilst HF- and DTM-SCS shift this balance towards an anti-inflammatory state. This observation is in line with the findings reported in a recent review and the discussed papers.^13,15,20,21^ It should however be taken into account that the pattern of the inflammatory balance as presented in Figure 5 is based on a limited number of makers: only two anti-ant two pro-inflammatory markers were included in this analyses. Although these markers were carefully selected based on their pivotal role in both the onset as well as maintenance of neuropathic pain, important contributions and impact of other cytokines cannot be ruled out.^28,29^ A limitation to our study is likely to be a dilution effect related to the dissectioning of the complete lumbar segment (L1-L6) rather than specific segments L4 and L5 only. Nevertheless, the macroscopical dissection of the complete lumbar section allowed a quick removal, which is needed for fast and optimal processing. The complete lumbar segment as we dissected includes L4 and L5 levels but a dilution effect on gene expression cannot be excluded.

In order to study the effects on glial cell activation, RNA expression of cell type markers for microglia (*Itgam*), astrocytes (*Gfap*), microglial proliferation markers *Irf8* and *Adgre1* and P2x4-MAPK pathway markers *P2x4*, *Mapk-14* and *Bdnf* were included in this study. Furthermore, RNA expression of T-cells (*Cd3d*) was included. The results indicate that 48h of Con-, HF- or DTM-SCS does not changes mRNA levels of microglial, astroglial or T-cell proliferation markers in a PDPN animal model. Interestingly, effects of Con-SCS have been reported on microglia and astroglia activation in PNI- induced models of chronic neuropathic pain.^23,24,47–49^ The difference in animal model may be important but as PDPN also includes damage to peripheral nerves, a similar glial response and mechanism might be expected. Induction of PDPN in animals without SCS was shown to induce an increase in microglial and astrocyte activity in the spinal dorsal horn, which is similar to that observed in studies using PNI injury models.^13,15,21,23–25,47–51^ In order to further understand the effect of PDPN a control group of naïve animals would have helped. Although the latter might be considered to be a limitation of this study it should be stressed that the aim of our study was to compare effects and mechanism of the various SCS paradigms on pain relief in PDPN. It is therefore that in view of this aim of the study we used parallel controls and this allowed to answer the research questions as addressed in the introduction. Again, another major difference between the studies using a PNI model and our study is the sex used. As described, in this PDPN-study only female animals were used, while in the studies using a PNI model only male animals were used.^13,15,21,23,24,47–51^ It has been described that in neuropathic pain the immune response may have dimorphic effects on males versus females.^33^ Therefore, we included a T-cell marker analysis. Nevertheless, neither of the SCS-paradigms used induced changes with respect to any of the cell type markers, including T-cells. This is remarkable as changes and effects on the inflammatory balance were present. More research is needed to investigate the relation between the inflammatory balance and cell activation in neuropathic pain and SCS. Furthermore, more insights in the distinct effects between males versus females on this inflammatory balance as related to SCS in neuropathic pain is needed. From our results it is not possible to directly demonstrate whether restoring the inflammatory balance by HF and DTM-SCS leads to less pain relief. However, our results can be a fundament for future pharmacological experiments using blockers or neutralizing antibodies investigating a causal effect.

In conclusion, spinal dorsal column stimulation with use of Con-, or HF- or DTM-SCS are equally effective at reducing mechanical hypersensitivity in STZ-induced PDPN animals after 24 and 48 h of continuous stimulation. No effects of either SCS paradigm is noted on motivational aspects of pain. Con-SCS resulted is a significant increase of *Tnf-α* and shows a shift of the inflammatory balance towards a pro-inflammatory state, whilst HF- and DTM-SCS shift the balance towards an anti-inflammatory state. These findings suggest that the underlying mechanism of Con-SCS induced pain relief in PDPN differs from that induced by HF- or DTM-SCS.
